# Impact of Analytical Treatment Interruption on Burden and Diversification of HIV Peripheral Reservoir: A Pilot Study

**DOI:** 10.3390/v13071403

**Published:** 2021-07-19

**Authors:** Rossana Scutari, Valentino Costabile, Laura Galli, Maria Concetta Bellocchi, Luca Carioti, Silvia Barbaliscia, Andrea Poli, Andrea Galli, Carlo Federico Perno, Maria Mercedes Santoro, Antonella Castagna, Francesca Ceccherini-Silberstein, Claudia Alteri, Vincenzo Spagnuolo

**Affiliations:** 1 Department of Experimental Medicine, University of Rome Tor Vergata, 00133 Rome, Italy; scutari.rossana@gmail.com (R.S.); mariac.bellocchi@gmail.com (M.C.B.); luca.carioti@yahoo.com (L.C.); s.barbaliscia@gmail.com (S.B.); santormaria@gmail.com (M.M.S.); ceccherini@med.uniroma2.it (F.C.-S.); 2 Department of Oncology and Hemato-Oncology, University of Milan, 20122 Milan, Italy; valentino.costabile@unimi.it; 3 Infectious Diseases, San Raffaele Scientific Institute, 20132 Milan, Italy; galli.laura@hsr.it (L.G.); poli.andrea@hsr.it (A.P.); galli.andrea@hsr.it (A.G.); castagna.antonella1@hsr.it (A.C.); spagnuolo.vincenzo@hsr.it (V.S.); 4 Unit of Diagnostic Microbiology and Immunology, Department of Laboratories, Bambino Gesù Children’s Hospital, IRCCS, 00165 Rome, Italy; carlofederico.perno@opbg.net; 5 Faculty of Medicine and Surgery, Vita-Salute San Raffaele University, 20132 Milan, Italy; 6 Multimodal Medicine Research Area, Bambino Gesù Children’s Hospital, IRCCS, 00165 Rome, Italy

**Keywords:** HIV-1, HIV-1 diversification, analytical treatment interruption, HIV-1 reservoir

## Abstract

Background: If analytical antiretroviral-treatment (ART) interruption (ATI) might significantly impact quantitative or qualitative peripheral-total HIV-DNA is still debated. Methods: Six chronically HIV-1 infected patients enrolled in APACHE-study were analysed for peripheral-total HIV-DNA and residual viremia, major-resistance-mutations (MRMs) and C2-V3-C3 evolution at pre-ATI (T1), during ATI (T2) and at achievement of virological success after ART-resumption (post-ATI, T3). These data were obtained at three comparable time-points in five chronically HIV-1 infected patients on suppressive ART for ≥1 year, enrolled in MODAt-study. Results: At T1, APACHE and MODAt individuals had similar peripheral-total HIV-DNA and residual viremia (*p* = 0.792 and 0.662, respectively), and no significant changes for these parameters were observed between T1 and T3 in both groups. At T1, 4/6 APACHE and 2/5 MODAt carried HIV-DNA MRMs. MRMs disappeared at T3 in 3/4 APACHE. All disappearing MRMs were characterized by T1 intra-patient prevalence <80%, and mainly occurred in APOBEC3-related sites. All MRMs persisted over-time in the 2 MODAt. C2-V3-C3 genetic-distance significantly changed from T1 to T3 in APACHE individuals (+0.36[0.11–0.41], *p* = 0.04), while no significant changes were found in MODAt. Accordingly, maximum likelihood trees (bootstrap > 70%) and genealogical sorting indices (GSI > 0.50 with *p*-value < 0.05) showed that T1 C2-V3-C3 DNA sequences were distinct from T2 and T3 viruses in 4/6 APACHE. Virus populations at all three time-points were highly interspersed in MODAt. Conclusions: This pilot study indicates that short ATI does not alter peripheral-total HIV-DNA burden and residual viremia, but in some cases could cause a genetic diversification of peripheral viral reservoir in term of both MRMs rearrangement and viral evolution.

## 1. Introduction

Antiretroviral therapy (ART) can suppress HIV-1 viremia below the detection limit in clinical assays tests and therefore is able to drastically reduce morbidity and mortality derived from HIV-1 infection [1,2]. Despite the success of ART, HIV cure cannot be achieved by antiretroviral therapy due to the persistence of proviral HIV-DNA in target cells. The HIV-DNA represents a latent reservoir that demonstrates remarkable stability [3,4]. Several studies reported that after ART initiation HIV-DNA burden drastically decreases during the first year of treatment, but afterwards the decay rate remains slow, and the HIV-DNA levels remain almost stable for a long-time [5,6,7].

To date multiple strategies aimed at preventing and/or contain the establishment and size of persistent HIV reservoirs have been applied [8,9,10]. Examples of such strategies include early initiation of ART, eliminating infected CD4^+^T-cells with therapeutic agents that target the virus and/or infected cells, and harnessing anti-HIV immune-responses using therapeutic vaccines or immunotherapies [11,12]. Early treatment can contribute to the reduction of residual viral replication and HIV reservoir size [13], can limit viral diversity, preserve innate immunity and lymphocyte functions [14,15,16], and contributing to HIV post-treatment control [17].

Recently, analytical treatment interruptions (ATI) or treatment simplification strategies have become a focus of investigations as innovative approaches to the long-term management of HIV disease. ATIs have been used in different studies towards an HIV cure, as in acute, primary and chronic infection [18,19] and in immune-based interventions [11,20]. The Visconti cohort clearly showed that early and prolonged cART may allow some individuals to gain long-term infection control, even during treatment interruption, paving the way for a drug free longterm control [15].

Current challenges with ATI include identifying an appropriate timeframe for length of interruptions and identifying HIV patient populations able to spontaneously control viral replication once antiretroviral drug therapy is withdrawn.

Nonetheless, concerns remain about the effect of ATI on the HIV-1 reservoir, and on the existence of reliable viral markers able to predict reservoir alterations during/post-ATI [21,22,23,24,25].

Recent data suggest that even in patients characterized by long period of HIV viral-load suppression and low viral reservoir (APACHE cohort), early and consistent viral-rebound can be observed during ATI [26]. Using samples previously collected for the APACHE study [26], we performed quantitative and qualitative analyses on peripheral HIV reservoir, to determine the effect of treatment interruption and transient viremia on the size and composition of this HIV compartment. All these patients fulfilled the criteria established by Julg et al. [27], for the inclusion in ATI protocols.

## 2. Materials and Methods

### 2.1. Study Population

This pilot study includes six chronically HIV-1 infected patients, enrolled in APACHE study (NCT03198325, experimental-group), who underwent ATI. These patients had HIV-RNA <50 copies/mL for ≥10 years, no viral blip for ≥5 years, CD4^+^T-cells >500 cells/mm^3^, HIV-DNA <100 copies/10^6^ PBMCs by Real-Time PCR (ABI-Prism 7900, Applied Biosystem, Foster City, CA, USA) and without comorbidities or AIDS-defining diseases. No patient initiated antiretroviral treatment during acute HIV-1 infection. We analyzed samples for peripheral-total HIV-DNA, residual viremia and HIV variability at three time-points: before ATI (pre-ATI, T1), at viral-rebound (VR) during ATI (defined as occurrence of two consecutive HIV-RNA >50 copies/mL on cART interruption, measured 2 weeks apart, T2) and at achievement of undetectable viral-load after ART resumption (post-ATI, T3; Figure S1A).

The same data were obtained at three comparable time-points (at baseline [T1], 12 weeks after T1 [T2], 12 weeks after T2 [T3]) in five chronically HIV-1 infected patients treated with a triple-therapy (triple-ART) and on stable virological suppression for >1 year, enrolled in the MODAT study (NCT01511809, control-group) [28] (Figure S1B).

Antiretroviral treatment of APACHE and MODAt individuals did not include drugs belonging to integrase inhibitor class (INI).

Peripheral blood mononuclear cells (PBMCs) aliquots and plasma samples were collected at all time-points.

For all patients, demographic and clinical information such as age, gender, CD4^+^T and CD8^+^T cell count were retrieved and stored in an anonymous database.

Ethical approval for the APACHE and the MODAt studies were obtained from the Ethical Committee of the San Raffaele (protocol number and approval date: 31/OSR on 17 May 2016 and 332/DG on 26 October 2010, respectively). Signed informed consent was obtained by all participants. The APACHE and the MODAt studies were conducted in accordance with the Declaration of Helsinki Ethical Principles and Good Clinical Practices.

### 2.2. Total HIV-DNA and Residual Viremia Quantification

Total HIV-DNA was quantified from a pellet of 3 × 10^6^ PBMCs by means of the QX200™ Droplet Digital™PCR System (ddPCR, Bio-Rad, Hercules, CA, USA) using home-made protocol targeting the LTR-5′ region [6]. Total HIV-DNA (copies/10^6^ PBMCs) was then normalized into number copies/10^6^ CD4^+^T-cells. Residual viremia was quantified from 2 mL of plasma through ddPCR using a home-made protocol targeting Integrase region [6].

### 2.3. Viral Sequencing

Genomic DNA was extracted from a pellet of 3 × 10^6^ PBMCs using DNeasy Blood & Tissue kit (Qiagen, Hilden, Germany). Plasma HIV-RNA was extracted using QIamp Viral RNA mini kit (Qiagen). Deep sequencing of POL (HXB2-pol nucleotides:170–1415) and C2-V3-C3 (HXB2-env nucleotides:805–1224) was performed from PBMCs and plasma on MiSeq platform (Illumina, San Diego, CA, USA). Briefly, HIV-DNA and HIV-RNA were amplified by PCR with region-specific primers (Table S1). Afterwards, five amplicons were obtained for POL and one amplicon for C2-V3-C3 by a modified protocol using AmpliTaq-Gold DNA-Polymerase (ThermoFisher, Waltham, MA, USA).

Subsequently to purification by Agencourt AMPure XP (Beckman Coulter, Brea, CA, USA) and quantification by Qubit dsDNA HS-Assay (ThermoFisher), amplicons were pooled in an equimolar concentration and indexed by Kapa-HiFi HotStart (Sigma-Aldrich, St. Louis, MO, USA). Lastly, 15 pM of denatured pool was sequenced using MiSeq-reagent kit v3 (2 × 300 base-pairs; Illumina) paired end run.

All sequences were screened for evidence of baseline quality control for short and low-quality sequences. A quality control was performed by USEARCH commands fastq_filter (using an accepted phred-score of 30) [29] and unoise3 (that remove chimera sequences through UCHIME-algorithm) [30]. The mapping of cleaned reads was performed against the GenBank reference genome HXB2 (K03455.1) using BWA-mem [31]. The mean coverage depth was 30,000× for both POL and C2-V3-C3. Reads mapping on HIV-1 reference genome were able to cover 1043 nucleotides corresponding to 71.9% of PR+RT (HXB2-pol nucleotides: 169–1618) and 230 nucleotides corresponding to 41.5% of C2-V3-C3 (HXB2-env nucleotides: 598–1152), independently by HIV-1 subtype. All single nucleotide polymorphisms having a minimum supporting read frequency of 2% with a depth ≥10,000 were retained. QuasiRecomb Software was used for haplotype reconstruction [32].

### 2.4. Estimation of Major Drug Resistance Mutations and Hypermutated Pol Sequences

Identical POL sequences (homology > 99%, number of sequences > 10) were clustered, allowing identification of most representative strains for each patient at each time point. The output consisting of a list of representative clusters of sequences and their relative frequencies was used to evaluate the presence of major drug resistance mutations (MRMs), hypermutant and thus defective strains, and stop codons.

MRMs to NRTI, NNRTIs or PI classes were defined according to the Stanford HIV Drug Resistance Database 2020 (https://hivdb.stanford.edu/, last update 23 October 2020, mutations reported in bold). INI resistance mutations were not considered for this analysis as patients were INI-naïve.

Wilcoxon signed-rank and Mann-Whitney test were used to estimate changes in MRMs prevalence within and between APACHE and MODAt subjects, respectively.

In order to define a trend of hypermutation and to detect potentially POL defective variants, G-to-A mutation frequencies at GA or GG POL sites potentially induced by APOBEC3-enzymes [33] and number of stop codons were predicted by https://www.hiv.lanl.gov/content/sequence/HYPERMUT/hypermut.html (accessed on 25 June 2021) using clustered and representative POL variants [34]. *p*-values were estimated for each sequence at each time-point by using as reference both HXB2 and the most abundant strain for each time-point. A *p*-value < 0.05, confirmed by using as reference both HXB2 and most abundant strain, was used to determine if a specific strain is a hypermutant, and thus probably defective.

### 2.5. Phylogenetic Analysis and Genealogical Sorting Index (GSI)

To assess potential compartmentalization among sampled virus populations, genetic distance and phylogenetic estimates were performed on C2-V3-C3 region.

Firstly, for each sample at each time-point, pairwise genetic distance was computed to quantify nucleotide diversity under the Tamura-Nei 93 model in the total number of obtained C2-V3-C3 strains [35]. Thus, evolution of viral diversity was evaluated between different time-points or over time within the same group (by Wilcoxon signed-rank or Friedman-test), or between groups at different time-points (Mann-Whitney test). Cohen’s delta coefficient was applied to exclude potential effect size (0 < |d| < 0.2 = negligible; 0.2 < |d| < 0.5 = small; 0.5 < |d| < 0.8 = medium; |d| > 0.8 = large).

To assess potential compartmentalization among sampled virus populations, identical C2-V3-C3 sequences (homology > 99%, number of sequences > 10) were clustered, allowing identification of most representative strains for each patient at each time point, following the procedure used for POL sequences. A maximum likelihood tree by using the combinations of clustered C2-V3-C3 proviral-DNA and viral-rebound sequences were inferred using PhyML version 3.0. To assess the relatedness of virus compartments more objectively, we determined genealogical sorting indices (GSI), that tests the phylogenetic similarities of 2 or more sequence groups, with the null hypothesis that the virus populations were the same [36]. GSI values range from 0 (complete interspersion) to 1 (complete monophyly), with statistical significance indicating greater than random segregation between/among groups. GSI values were calculated using the genealogical Sorting R package (http://molecularevolution.org/software/phylogenetics/gsi) (accessed on 21 November 2020) [36]. Statistical significance was assessed by randomly permuting character states across the tips of the tree 1000 times. No significant values indicated the absence of compartmentalization.

All *p*-values were two-sided and corrected for multiple tests (Benjamini-Hockberg correction) [37]. A *p*-value of less than 0.05 was considered statistically significant. All analyses were performed using statistical software Rgui and SPSS version 19 (IBM Corp. Armonk, NY, USA).

## 3. Results

### 3.1. Patients’ Population

The demographic and clinical characteristics by APACHE and MODAt participants are shown in Table 1. Patients were mainly male and infected by B subtype (8/11; 72.7%). At study entry, patients were on suppressive ART (median (Interquartile-range, IQR) residual viremia: 2 (2–5) vs. 3 (2–7) copies/mL in APACHE and MODAt, respectively; *p*-value = 0.662) for more than 2 years (median [IQR]: 11.5 [11.0–11.9] vs. 4.7 [2.9–6.7] in APACHE and MODAt, respectively; *p*-value = 0.008). APACHE individuals experienced viral-rebound between 14 and 56 ATI days and restarted ART after confirmation of two viremia determinations >50 copies/mL. APACHE plasma viral-load was again <50 copies/mL after 95 days (IQR:19–168) of ART restart (median (IQR) residual viremia: 4 (1–9) copies/mL).

MODAt participants remained under virological control for all the study period.

### 3.2. Quantitative Measures of Reservoir Change

To estimate within-person changes in the size of the peripheral latent reservoir pre- and post-ATI, we quantified peripheral-total HIV-DNA from PBMCs by ddPCR for all participants at different time-points.

Firstly, no significant differences were observed in peripheral-total HIV-DNA values at T1, T2, and T3 between APACHE and MODAt subjects. In particular, median (IQR) peripheral-total DNA levels was 1048 (863–1286) and 1037 (730–1750) copies/10^6^ CD4^+^T-cells at T1 (pre-ATI), and 1916 (398–3798) and 1087 (663–1567) copies/10^6^ CD4^+^T-cells at T2, in APACHE and MODAt subjects, respectively. At T3, the median (IQR) HIV-DNA was 1333 (553–2183) in APACHE-group and 1104 (527–1598) in the MODAt-group.

Regarding change in time, time between T2-T1 was significantly lower in APACHE respect to MODAt participants (median [IQR] weeks: 3.4 [2.9–5] vs. 12.0 [11.9–12.0], respectively; *p*-value = 0.004). No difference was detected at T3-T2 (median [IQR] weeks: 18.6 [4.4–28.6] vs. 12.0 [12.0–12.1], respectively; *p*-value = 0.537).

**Figure 1:**
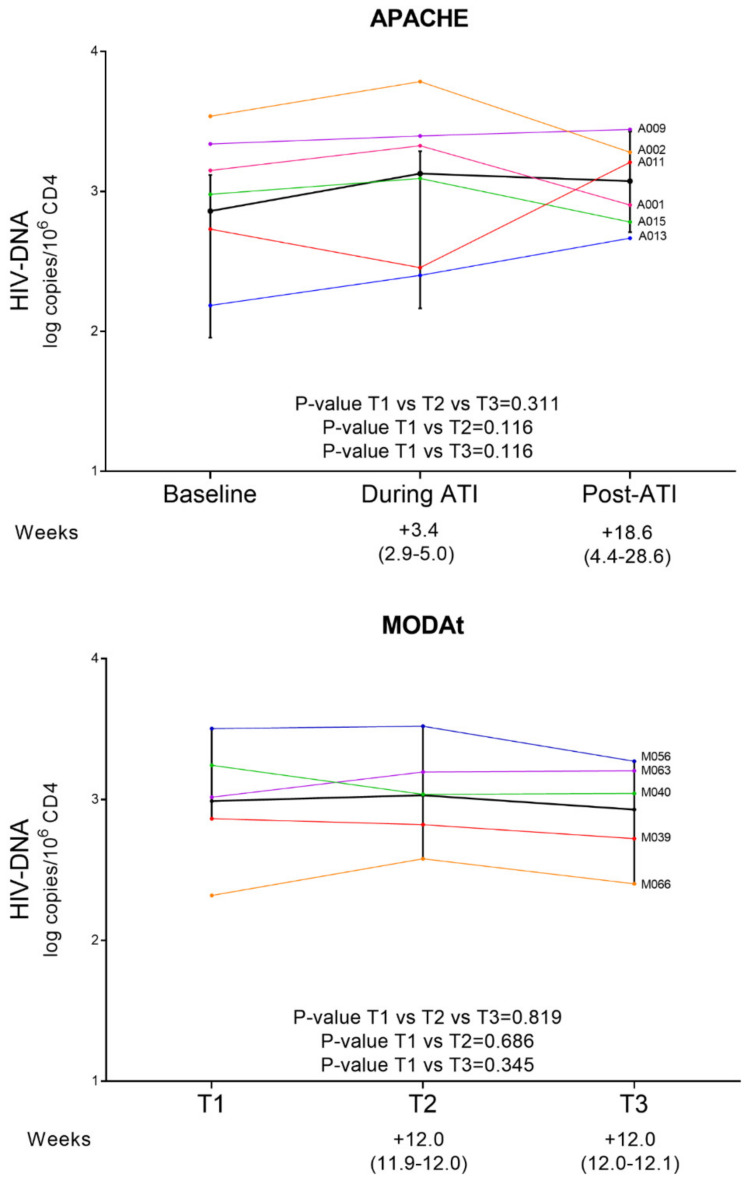
Total HIV-DNA of APACHE and MODAt patients at T1, T2 and T3. Each line represents a patient. The black line represents the mean value at each time point, accompanied by error bars. Table 1 to T2 and from T2 to T3 is expressed in weeks (median, IQR). *p*-value T1 vs. T3: Wilcoxon-test; *p*-value T1 vs. T2 vs. T3 Friedman tests.

Looking at HIV-DNA at different time-points, transient, but not significant, increase of HIV-DNA during ATI was found in APACHE subjects (*p*-value = 0.311, Figure 1), while a stable HIV-DNA values over-time was detected in MODAt (*p*-value = 0.819, Figure 1). This is confirmed by comparing the decrease in HIV-DNA at different time points in APACHE and MODAt participants (Figure S2). In detail, in APACHE individuals a positive ΔT2–T1 HIV-DNA (median [IQR]: 0.44 [0.17; 0.65]) and a negative ΔT3-T2 HIV-DNA (median [IQR]: −0.19 [−0.53; 0.40]) were observed, underlining the increase of HIV-DNA at viral rebound and then a restoration at T3. On the other hand, in MODAt subjects the Δ values are almost comparable at different time points (median [IQR]; ΔT2–T1: 0.02 [−0.04; 0.18], ΔT3–T2: −0.09 [−0.18; 0.01], ΔT3–T1: −0.14 [−0.20; 0.08]) thus indicating a stability of HIV-DNA over time.

To further evaluate if the modification of HIV-DNA levels observed in APACHE individuals might be affected by the duration in weeks between time-points, ΔHIV-DNA (T2–T1 HIV-DNA and T3–T2 HIV-DNA, respectively) were compared against Δweeks (T2–T1 weeks and T3–T2 weeks, respectively). Of note, longer was the change in time at T3–T2, higher was the change in HIV-DNA observed at T3–T2 (Rho = 0.829, *p*-value = 0.042), confirming the decay of HIV-1 reservoir against time [5,6,7]. No significant correlation was observed between the T2-T1 HIV-DNA change and the change in time between T2 and T1 (Rho = 0.464, *p*-value = 0.354).

### 3.3. Major Drug Resistance Mutations Prevalence According to Time Points

At T1, 4/6 APACHE and 2/5 MODAt subjects carried HIV-DNA MRMs. The median intra-patient prevalence was 38.0% (IQR: 8.9–74.8) (mutational load: 249 [81–920] copies/10^6^ CD4^+^T-cells) for APACHE, and 58.2% (IQR: 47.1–99.5) (mutational load: 726 (179–824) copies/10^6^ CD4^+^T-cells) for MODAt individuals (Table 2).

At T2, HIV-DNA MRMs persisted in 2/4 (50.0%) APACHE individuals, with an intra-patient prevalence that decreased from pre-ATI to ATI (38.0% [8.9–74.8] vs. 10.8% [5.0–16.2], *p*-value = 0.06; mutational load: 250 [81–920] vs. 65 [0–409] copies/10^6^ CD4^+^T-cells, *p*-value = 0.017).

At T3, HIV-DNA MRMs persisted only in 1 out 4 APACHE individuals (25.0%, Table 2, patient A001). Of note, this patient was the only one presenting several MRMs with a baseline intra-patient prevalence >80%, suggesting the presence of a dominant resistance viral species. In the other APACHE patients, the archived MRMs completely disappeared (intra-patient prevalence: 17.4% [6.2–38.0] vs. 0.0% [0.0–0.0], *p*-value = 0.008; mutational load: 156 [67–277] vs. 0 [0–0] copies/106 CD4^+^T-cells, *p*-value = 0.008).

In the MODAT-group, MRMs persisted at DNA level in both subjects with baseline MRMs at both T2 and T3 (ID: M039, M040; Table 2).

### 3.4. Defective Mutants over Time

Hypermutated sequences (*p*-value < 0.05) were found only in 1/6 APACHE sample (ID: A009) (*p* = 0.002 by HXB2 and *p*-value = 0.0005 by most abundant strain; intra-patient prevalence: 10.7%, time = T1). Of note, the two APOBEC3-related mutations at drug-resistance positions characterizing this patient (D67N: 6.2% and E138K: 7.3%) were present in the hypermutant POL strains, thus suggesting the presence of this mutation in potentially defective proviruses. Accordingly, these variants were never detected at following time-points (T2 and T3). Interestingly, at T1 other 3 APACHE individuals carried APOBEC3-related mutations at drug-resistance positions (intra-patient prevalence: median 6.8% [IQR: 5.7–28.2], Table 2). In MODAt individuals, APOBEC3-related mutations were present only in 2/5 patients (M039 at T2 and M063 at T3) and never with an intra-patient prevalence higher than 10%.

In line with the low frequency of hypermutated strains, stop-codons were rarely found in both APACHE and MODAt patients, and always with an intrapatient prevalence below the 8%, with the sole exception of Q23 * (at T2 of A015; intra-patient prevalence: 71%) (Table 1).

### 3.5. C2-V3-C3 Evolution

**Figure 2:**
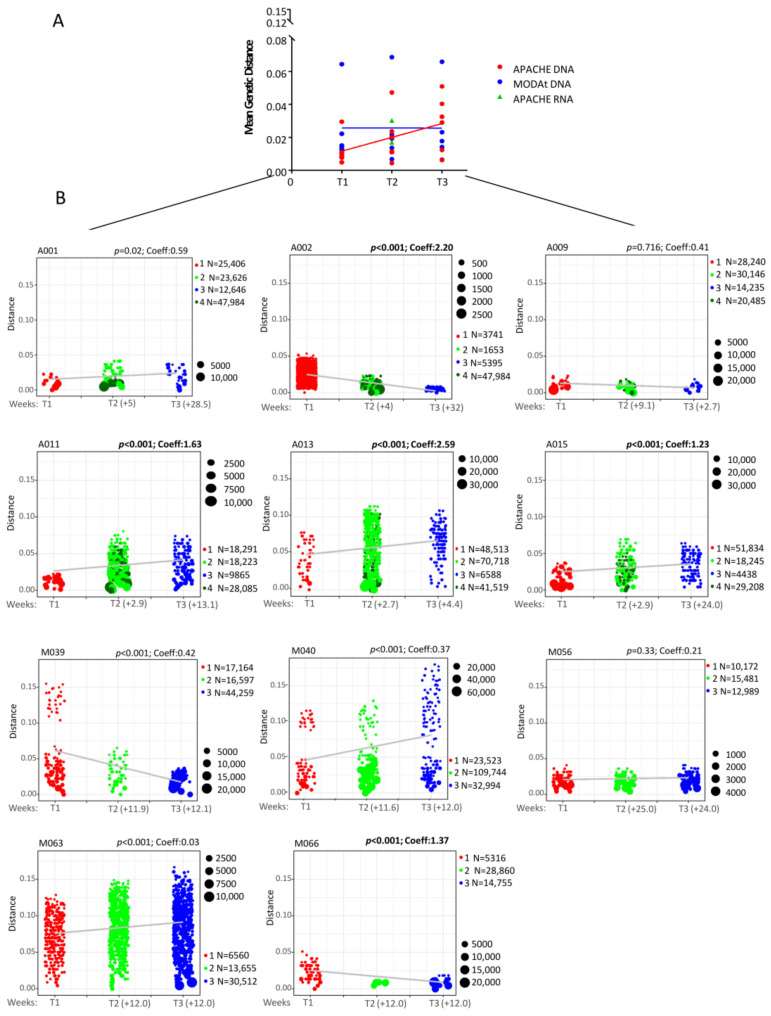
Change in HIV DNA molecular diversity (**A**) and C2-V3-C3 pairwise distance estimated for all samples in APACHE and MODAt individuals (**B**). (**A**) Mean pairwise distance was estimated for all samples within C2-V3-C3 region in APACHE (red dots for DNA and green dots for RNA) and MODAt (blue dots) individuals. For each sample, the means of all pairwise Tamura-Nei 93 (TN93) distances among sequences were computed to quantify nucleotide diversity. *p*-value T1 vs. T3: Wicoxon-test; *p*-value T1 vs. T2 vs. T3 Friedman tests. (**B**) For each sample, the means of all pairwise Tamura-Nei 93 (TN93) distances among sequences was defined to quantify nucleotide diversity. *p*-value T1 vs. T3: Mann-Whitney test and Cohen’s delta coefficient. Samples with a C2-V3-C3 pairwise distance characterized by a Cohen’s delta coefficient >0.8 and by a *p*-value < 0.05 were shown in bold. The time (weeks) from T1 to T2 and from T2 to T3 were reported for each patient. The black dots represent the abundance, Ns represent the numbers of sequences.

In order to define the viral evolution in APACHE and MODAt individuals, a pairwise distance within C2-V3-C3 region was firstly assessed (Figure 2A). In detail, by comparing C2-V3-C3 sequences, we observed a significant increase in overall genetic distance from T1 to T3 in APACHE individuals (median [IQR]: +0.036 [0.011–0.041], *p*-value = 0.04), while no significant difference was found in MODAt patients (+0.018 [−0.001–0.024], *p*-value = 0.22). These results were also confirmed by comparing with Friedman tests the C2-V3-C3 genetic distance at the three time-points (APACHE: T1 vs. T2 vs. T3, *p*-value = 0.04; MODAt: T1 vs. T2 vs. T3, *p*-value = 0.59). Moreover, by applying nonparametric tests to the longitudinally collected samples for each patient, a significant variation in genetic diversity from T1 to T3 was found for 4/6 APACHE individuals, with the sole exception of A001 and A009, and 1/5 MODAT individuals (*p*-value < 0.05 and Cohen’s delta coefficient >0.8, Figure 2B). Of note, A001 and A009 were characterized by high homogenous viral population at pre-ATI, as suggested by the low genetic variability in C2-V3-C3 (Choen’s coefficient: 0.59 and 0.41 respectively, Figure 2).

**Figure 3:**
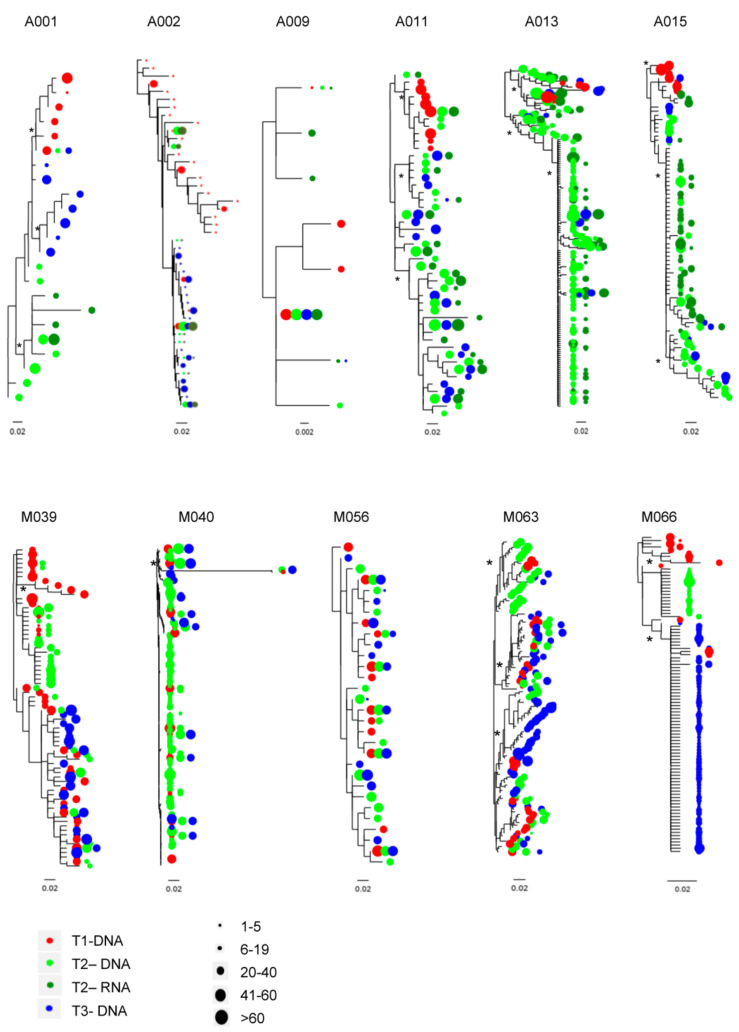
Maximum likelihood phylogenetic reconstruction of C2-V3-C3 region in APACHE (A) and MODAt (M) patients (PhyML). C2-V3-C3 sequences were used to construct maximum likelihood phylogenies. Time points 1, 2, and 3 are depicted in red, blue, and green, respectively. RNA at time 2 is depicted in dark green. Tip size is proportional to variant frequency. Scale bars are in substitutions/site. Asterisks (*) indicate a bootstrap ≥70%.

Subsequently, a C2-V3-C3 phylogenetic analysis was carried out on representative clustered strains to evaluate whether the treatment interruption and the transient viremia changed the composition of the populations of the viral reservoir. Maximum likelihood phylogenetic trees showed that in 5/6 (83%) APACHE patients pre-ATI DNA sequences were generally distinct (boostrap > 70%) from viral-rebound and post-ATI viruses (Figure 3), and that in vivo viral-rebound was not predicted by the expansion of pre-ATI viral lineage. Differently, post-ATI viruses were enriched by viral-rebound strains in 4/6 (66.7%) APACHE individuals, with the sole exception of A001 and A009. Of note, the lack of evolution observed at post-ATI in A001 and A009 is in line with the results obtained by MRMs evaluation and C2-V3-C3 genetic distance.

On the contrary, in 4/5 (80%) MODAt patients the phylogenies for C2-V3-C3 regions showed that virus populations at the three time-points were highly interspersed (Figure 3).

Furthermore, to assess the relatedness of virus compartments more objectively, we determined a genealogical sorting index (GSI). GSI values range from 0 (complete interspersion) to 1 (complete monophyly), with statistical significance indicating greater than random segregation between/among groups.

In APACHE patients, GSI values obtained at different time-points indicated that the majority of participants had highly monophyletic virus populations (GSI > 0.50; *p*-value < 0.05). Only the sequences for participants A001 and A009 had low GSI value, indicating similar sequences between T1 and T3 for A001 and highly interspersed sequences over-time for A009 (Table 3). Looking at pairwise comparisons, in 5 out of six participants, the T1 proviral sequences differed significantly (GSI > 0.50; *p*-value < 0.05) from T2 (both proviral and rebound viruses), suggesting that the rebound viruses during ATI does not arise from the pre-ATI sequences. In four out of six participants, both the T2 proviral and rebound sequences did not differ significantly (GSI ≤ 0.50; *p*-value < 0.05) from T3 proviral-DNA, suggesting a contribution of rebound sequences to viral evolution of post-ATI viruses.

Differently, in MODAt patients GSI values indicated that the majority of participants had highly interspersed virus populations (GSI ≤ 0.50; *p*-value > 0.05). Indeed, low and no significant GSI values between T1 and T3 were found in 80% (4/5) MODAT patients. Only patient M66 had a statistically significantly high GSI value, indicating changes in the composition of the proviral-DNA over-time (Table 3). The highly interspersed T1-, T2- and T3-sequence sets support the concept that a continuous viral suppression no substantial changes to the sampled reservoir.

## 4. Discussion

Today, a major objective of current HIV research is the development of functional cure strategies aimed to achieve long-term virological remission [24,38]. In this context, short-term ATI has been occasionally used, even if doubts and uncertainties about the role of ATI in the short term remain [19,23,26].

Here, we used samples from patients enrolled in the APACHE study [26] to assess the effect of ATI in term of burden and rearrangement of peripheral HIV reservoir, in well characterized subjects with common features, like plasma HIV-RNA <50 copies/mL for ≥10 years, no viral blip for ≥5 years, CD4^+^T-cells >500 cells/mm^3^, HIV-DNA <100 copies/10^6^ PBMCs and without comorbidities or AIDS-defining diseases. These patients were compared with a group of patients enrolled in the MODAt study (subjects in triple-ART in virological suppression) [28].

We firstly assessed the impact of ATI on the amount of peripheral-total HIV-DNA, considered as a global marker of HIV persistence and progression [39,40]. We did not find any significant change in the size of the peripheral reservoir between pre-ATI and post-ATI. Only a transient, but insignificant, increase in HIV-DNA during ATI was found in subjects belonging to APACHE study (*p*-value = 0.311, Figure 1). No differences in HIV-DNA were also found over-time among the MODAt participants. These results are in line with data published so far, that reported no significant change in the size of the HIV-1 peripheral reservoir after ART resumption [21,26], with the only exception for a transient expansion of HIV-1 reservoir in CD4^+^T-cells, as well as immune markers, during ATI [21].

Even if total HIV-DNA quantification reveals a stability in the amount of peripheral HIV reservoir, differences in the archived MRMs between pre- and post-ATI were found. In particular, in three out four APACHE individuals (ID: A002, A009, A015), pre-ATI MRMs were lost during virological rebound and in post-ATI HIV-DNA. All these MRMs were characterized by pre-ATI intra-patient prevalence <80%. The only APACHE individual where a consistent disappearance of MRMs was not observed (A001), was mostly characterized by MRMs with an intra-patient prevalence >80%. Overall data suggest that the absence of ART selective pressure during ATI combined with the low intra-patient prevalence of some MRMs might participate in the disappearance of low-abundant drug resistance mutations. In line with the hypothesis that ATI and the absence of ART selective pressure might cause a re-assortment of MRMs, in MODAt patients drug resistance mutations persisted across all three time-points independently by their baseline intra-patient prevalence.

Interestingly, most of mutations at drug resistance positions found in APACHE subjects (i.e., D67N, G190E, E138K, M184I) derived by G to A substitutions, and thus probably related to an hypermutation process induced by APOBEC3-enzymes, as suggested by the presence of D67N and E138K in hypermutant and probably defective HIV-DNA strains at T1 of A009. Thus, the presence of these mutations in potentially defected strains might have accelerated their disappearance at following time points [41].

No emergence of new MRMs was observed neither during ATI nor post-ATI, data in line with Clarridge and colleagues, who observed no evidence of emergence of antiretroviral drug resistance mutations within intact HIV proviral-DNA sequences following reinitiation of ART [21].

In line with the disappearance of MRMs observed in APACHE individuals during and after ATI, the analysis of C2-V3-C3 revealed a more pronounced viral evolution in APACHE than in MODAt participants. In particular, a significant C2-V3-C3 divergence was observed from T1 to T3 in the majority of APACHE subjects and in none of MODAt individuals. In parallel with the variation in genetic distance, phylogenetic analyses showed significant differences in the composition of the peripheral reservoir before and after ATI in APACHE individuals, and no substantial differences in MODAt patients. In particular, the pre-ATI HIV-DNA strains did not align closely with the rebound viruses in most of the APACHE participants (5/6). The striking discordance between the pre-ATI sequences and rebound viruses can be explained by a fraction of a potentially large and diverse reservoir capable of reactivating, and probably arisen by different HIV reservoir (i.e., lymph nodes or central nervous system or GALT). These results are in accordance with Salantes and colleagues, that through phylogenetic analyses of HIV-1 env sequences from QVOA and proviral-DNA, showed that pre-ATI reservoir sampling did not predict viral-rebound [22]. Differently by Salantes and colleagues that showed a minimal viral diversification between pre-ATI and post-ATI, in our population viral strains found in post-ATI appeared to be enriched by ATI in four out of six individuals, suggesting that transient viremia could partially influence the genetic divergence of the peripheral reservoir [25]. Only in two participants (A001 and A009) the presence of a dominant probably clonally expanded population in pre-ATI sampling contributes to post-ATI sequences.

Notably, the apparently different results between Salantes and us can be explained by the distinct populations analysed in the two studies. In our work, the timing of the sampling post-ATI is just at achievement of undetectable VL after ART resumption, and not at least 6 months after viral suppression [11,22]. This might explain the higher genetic imprint of viral rebound variants in the post-ATI reservoir repertoire found in our patients. Moreover, the study by Salantes and colleagues, based on single genome sequencing approach, includes individuals who underwent ATIs after receiving the infusion of the bnAb VRC01 [11]. This intervention may have had consequences on the HIV reservoir, by enhancing its stability. Differently, our study is characterized by individuals who underwent ATIs without any other intervention, allowing us to appreciate the role of ATIs devoid of any kind of interference.

Differently by APACHE individuals, the highly interspersed C2-V3-C3 sequences at the different time-points in MODAt participants support the hypothesis that in presence of continuous viral suppression no substantial changes to the sampled reservoir can occur.

Our study has some limitations. We did not evaluate replication-competent virus neither cell-associated HIV-RNA, defined as a biomarker of HIV persistence and latency reversion. The amount and the characterization of viral reservoir in other districts, like lymph nodes, GALT, was not assessed. In order to define viral evolution, we analysed pol and C2-V3-C3 region, and not the whole HIV-1 genome, even if all the analyses provided concordant and reliable results. Due to the conservative criteria we adopted for ART resumption, we could not evaluate the immune-mediated viral control that could theoretically have occurred after an initial transient rebound. Finally, the small number of patients here analyzed limits the power of the study and the possibility to draw general conclusions. Detecting differences between individuals who underwent ATI versus those who successfully continued ART in the considered outcomes, or their determinants remains challenging.

## 5. Conclusions

In conclusion, our results confirm that a closely monitored short interruption of treatment does not affect the amount of post-ATI peripheral-total HIV-DNA values, nor the efficacy of ART in inducing viral suppression, thus representing a safe intervention. In some patients, it might cause a genetic diversification of peripheral viral reservoir both in term of MRMs rearrangement and in term of viral evolution, that again do not affect the efficacy of antiretroviral drugs used. These results do not exclude that rebound viral strains released from different anatomical HIV reservoirs (i.e., GALT or lymph-nodes or CNS) might participate in the modification of new peripheral viral composition of post-ATI reservoir. In two cases ATI did not cause peripheral virus rearrangement. Both cases presented reduced viral divergence at T1 as suggested by the limited C2-V3-C3 intra-host variability and the presence of fixed resistant mutations in the almost totality viral population. Thus, we can speculate that the presence of a dominant population at baseline of treatment interruption might be related with lower probability of peripheral reservoir rearrangement during/post-ATI. No patient enrolled in this study achieved long-term virological control in the absence of ART, thus limiting the possibility to draw conclusions about parameters related to HIV control. Thus, a complete definition of key and reliable viral parameters for future HIV-1 cure strategies remains challenging.

## Figures and Tables

**Table 1:** Patients’ characteristics.

Patients’ Characteristics	Overall (N = 11)	APACHE Study (N = 6)	MODAt Study (N = 5)	*p*-Value ^§^
Age (years), median (IQR)	47.3 (43.4–1.2)	49.5 (47.3–51.2)	44.1 (43.4–45.2)	0.235
Male gender, N (%)	8 (72.7)	5 (83.3)	3 (60.0)	0.545
Subtype B, N (%)	8 (72.7)	3 (50.0)	5 (100.0)	0.182
Years since first HIV positive test, median (IQR)	15.3 (9.3–19.4)	17.1 (15.3–22.8)	9.3 (4.0–11.4)	0.055
Years since ART start, median (IQR)	11.1 (6.1–17.6)	17.1 (13.5–18.1)	6.1 (3.2–9.0)	**0.008**
Years of HIV-1 RNA <50 copies/mL, median (IQR)	9.6 (4.7–11.7)	11.5 (11.0–11.9)	4.7 (2.9–6.7)	**0.008**
Viro-immunological parameters at T1 (median, IQR):				
HIV-DNA (copies/10^6 ^CD4^+^T-cells)	1037 (730–1311)	1048 (863–1286)	1037 (730–1750)	0.792
HIV-RNA * (copies/mL)	2 (2–7)	2 (2–5)	3 (2–7)	0.662
CD4^+^T-cells (cells/mm^3^)	748 (563–1012)	737 (563–1512)	774 (745–777)	0.931
CD8^+^T-cells (cells/mm^3^)	814 (451–1203)	1017 (333–1235)	685 (534–814)	0.523
CD4^+^/CD8^+^ ratio	1.24 (0.72–2.04)	1.44 (0.61–2.15)	1.24 (0.82–1.46)	0.784
Viro-immunological parameters at T2 (median, IQR):				
HIV-DNA (copies/10^6^ CD4^+^T-cells)	1567 (398–3307)	1916 (398–3798)	1087 (663–1567)	0.537
HIV-RNA (copies/mL)	2984 (3–69,820)	60,108 (12,251–1,680,510)	3 (2–4) *	**0.004**
CD4^+^T-cells (cells/mm^3^)	731 (521–1004)	590 (497–1064)	765 (731–890)	0.537
CD8^+^T-cells (cells/mm^3^)	829 (454–1341)	1041 (279–1341)	750 (594–1296)	0.998
CD4^+^/CD8^+^ ratio	1.19 (0.54–1.46)	1.13 (0.53–1.78)	1.19 (0.77–1.29)	0.784
Viro-immunological parameters at T3 (median, IQR):				
HIV-DNA (copies/10^6^ CD4^+^T-cells)	1104 (527–1867)	1333 (553–2183)	1104 (527–1598)	0.537
HIV-RNA * (copies/mL)	3 (0–7)	4 (1–9)	0 (0–5)	0.329
CD4^+^T-cells (cells/mm^3^)	816 (645–1096)	987 (645–1134)	728 (728–816)	0.429
CD8^+^T-cells (cells/mm^3^)	1003 (523–1257)	1066 (491–2058)	1003 (744–1043)	0.783
CD4^+^/CD8^+^ ratio	0.87 (0.62–1.33)	1.06 (0.51–1.33)	0.85 (0.78–0.98)	0.927

^§^ *p*-values by Mann-Whitney test or chi-square/Fisher exact test, as appropriate. * Residual viremia. Statistically significant differences (*p*-values < 0.05) are highlighted in bold.

**Table 2:** Major drug resistance mutations and stop-codons in protease and reverse transcriptase found in APACHE and MODAT participants at different time points.

Study	ID	Time 1	Time 2		Time 3
APACHE	Sample	HIV-DNA	HIV-DNA	HIV-RNA	HIV-DNA
	A001	**PR:** None **RT: D67N** (99.4%), K70R (99.6%), M184V (14.0%), T215I (85.6%), K219Q (99.8%)	**PR:** None **RT: D67N** (21.3%), K70R (99.7%), M184V (11.1%), K219Q (10.2%)	**PR:** None **RT:** K70R (99.6%)	**PR:** None **RT: D67N** (99.3%), K70R (99.7%), M184V (8.1%), T215I (91.9%), K219Q (99.8%)
	A002	**PR:** None **RT:** W88 *(5.0%), **G190E** (5.1%), W212 *(5.0%)	**PR:** None **RT:** W88 *(5.0%), W212 *(7.0%)	**PR:** None **RT:** W88 *(5.0%), W212 *(7.0%)	**PR:** None **RT:** None
	A009	**PR:** None **RT: D67N** (6.2%), **E138K** (7.3%), W153 * (4.8%), W212 * (8.5%)	**PR:** None **RT:** None	**PR:** None **RT:** None	**PR:** None **RT:** None
	A011	**PR:** None **RT:** None	**PR:** None **RT:** None	**PR:** None **RT:** None	**PR:** None **RT:** None
	A013	**PR:** None **RT:** None	**PR:** None **RT:** None	**PR:** None **RT:** None	**PR:** None **RT:** None
	A015	**PR:** None **RT:** K65R (39.5%), K101E (38.0%), Y181C (40.2%), **M184I** (28.9%)	**PR:** None **RT:** Q23 * (71.0%), K65R (3.5%), Y181C (6.5%), **M184I** (3.6%), W153 *(5.0%)	**PR:** None **RT:** None	**PR:** None **RT:** None
MODAt	Sample	HIV-DNA	HIV-DNA		HIV-DNA
	M039	**PR:** None **RT:** M41L (99.6%), T215C (99.5%)	**PR:** None **RT:** M41L (99.6%), **M184I** (6.9%), T215C (99.4%)		**PR:** None **RT:** M41L (93.7%), T215C (98.6%)
	M040	**PR:** None **RT:** M41L (47.1%)	**PR:** None **RT:** M41L (73.9%)		**PR:** None **RT:** M41L (31.7%)
	M056	**PR:** None **RT:** None	**PR:** None **RT:** None		**PR:** None **RT:** None
	M063	**PR:** W42 *(8.0%) **RT:** None	**PR:** None **RT:** None		**PR:** None **RT:** None
	M066	**PR:** None **RT:** None	**PR:** None **RT:** None		**PR:** W42 *(5.0%), **M46I** (5.1%) **RT:** W153 * (12.1%)

All the major drug resistance mutations (Stanford list 2020) detected in HIV-1 protease and reverse transcriptase by Illumina MiSeq with a prevalence >2% have been considered. APOBEC DRMs (Stanford list 2020): PR: D30N, M46I, G48S, G73S; RT: D67N, V106I, E138K, M184I, G190ES, M230I. APOBEC3-related MRMs are highlighted in bold. PR: protease; RT: reverse transcriptase. *: Stop codons.

**Table 3:** Genealogical Sorting Indices for C2-V3.

		T1 DNA vs. T2 DNA	T1 DNA vs. T2 RNA	T1 DNA vs. T3 DNA	T2 DNA vs. T2 RNA	T2 RNA vs. T3 DNA	T2 DNA vs. T3 DNA
APACHE	A001	1.00 * vs. 0.99 *	1.00 * vs. 0.99 *	0.50 vs. 0.99 *	0.11 vs. 0.32	1.00 * vs. 0.99 *	0.96 * vs. 0.98 *
APACHE	A002	0.16 vs. 0.68 *	0.32 vs. **0.72** *	0.61 * vs. 0.73 *	0.43 vs. 0.68 *	0.01 vs. 0.01	0.29 vs. 0.45
APACHE	A009	0.20 vs. 0.08	0.08 vs. 0.10	0.06 vs. 0.08	0.05 vs. 0.08	0.05 vs. 0.08	0.07 vs. 0.18
APACHE	A011	0.89 * vs. 0.52 *	0.93 * vs. 0.57 *	1.00 * vs. 0.99 *	0.11 vs. 0.16	0.48 vs. 0.44	0.42 vs. 0.46
APACHE	A013	0.85 * vs. 0.70 *	0.92 * vs. 0.99 *	0.94 * vs. 0.56 *	0.11 vs. 0.26	0.31 vs. 0.21	0.37 vs. 0.19
APACHE	A015	0.99 * vs. 0.84 *	0.93 * vs. 0.55 *	0.93 * vs. 0.55 *	0.28 vs. 0.31	0.90 * vs. 0.45	0.69 * vs. 0.47
MODAt	M039	0.62 * vs. 0.33	//	0.47 vs. 0.78 *	//	//	0.34 vs. 0.81 *
MODAt	M040	0.10 vs. 0.16	//	0.10 vs. 0.18	//	//	0.16 vs. 0.18
MODAt	M056	0.23 vs. 0.11	//	0.11 vs. 0.15	//	//	0.20 vs. 0.37
MODAt	M063	0.32 vs. 0.40	//	0.75 * vs. 0.51 *	//	//	0.38 vs. 0.37
MODAt	M066	1.00 * vs. 0.99 *	//	1.00 * vs. 0.95 *	//	//	1.00 * vs. 0.99 *

Genealogical-Sorting-Indexes (GSI) values were calculated using the genealogical Sorting R package. (http://molecularevolution.org/software/phylogenetics/gsi/download, accessed on 21 November 2020). GSI values range from 0 (complete interspersion) to 1 (complete monophyly). Statistical significance was assessed by randomly permuting character states across the tips of the tree 1000 times. Non-significant values indicate the absence of compartmentalization. GSI > 0.50 accompanied by *p*-value < 0.05 are in bold. * *p*-value < 0.05 after Benjamini-Hockberg correction.

## Data Availability

The data presented in this study are available on request from the corresponding author.

## Supplementary Materials

The following are available online at https://www.mdpi.com/article/10.3390/v13071403/s1, Figure S1: Time points and virological parameters analyzed in APACHE (**A**) and MODAt patients (**B**). Figure S2: HIV-DNA decrease at different time-points. The Δ HIV-DNA was calculated as the difference in HIV-DNA values at different time-points considered. *p*-values were estimated by Mann-Whitney test. Table S1: NGS primers used to sequencing HIV-1. Clustered POL and C2-V3-C3 sequences were submitted to GenBank and are available under the following accession numbers: POL BankIt2476731: MZ494751–MZ494949; V3 BankIt2476739: MZ494950–MZ49622.
