# Supplementary material for: Impact of Analytical Treatment Interruption on Burden and Diversification of HIV Peripheral Reservoir: A Pilot Study
Source: Viruses. 2021 Jul 19;13(7):1403. doi: 10.3390/v13071403 (PMC8310290; doi:10.3390/v13071403)
Supplement: Supplementary file 1 [file viruses-13-01403-s001.zip › SupplFigureS1.pptx]

## Slide 1
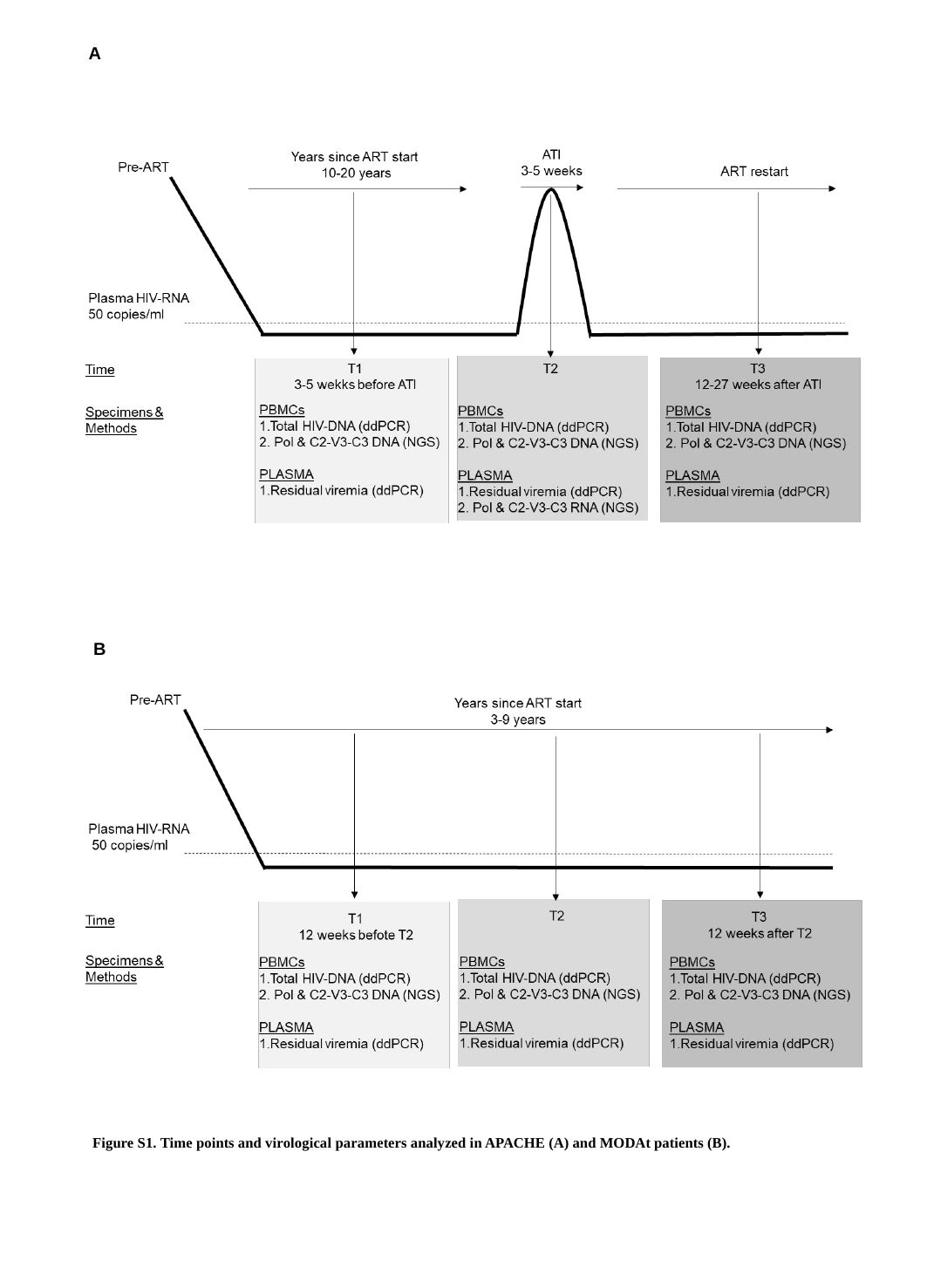

A
B
 Figure S1. Time points and virological parameters analyzed in APACHE (A) and MODAt patients (B).
