# Supplementary material for: Impact of Analytical Treatment Interruption on Burden and Diversification of HIV Peripheral Reservoir: A Pilot Study
Source: Viruses. 2021 Jul 19;13(7):1403. doi: 10.3390/v13071403 (PMC8310290; doi:10.3390/v13071403)
Supplement: Supplementary file 1 [file viruses-13-01403-s001.zip › SupplFigureS2.pptx]

## Slide 1
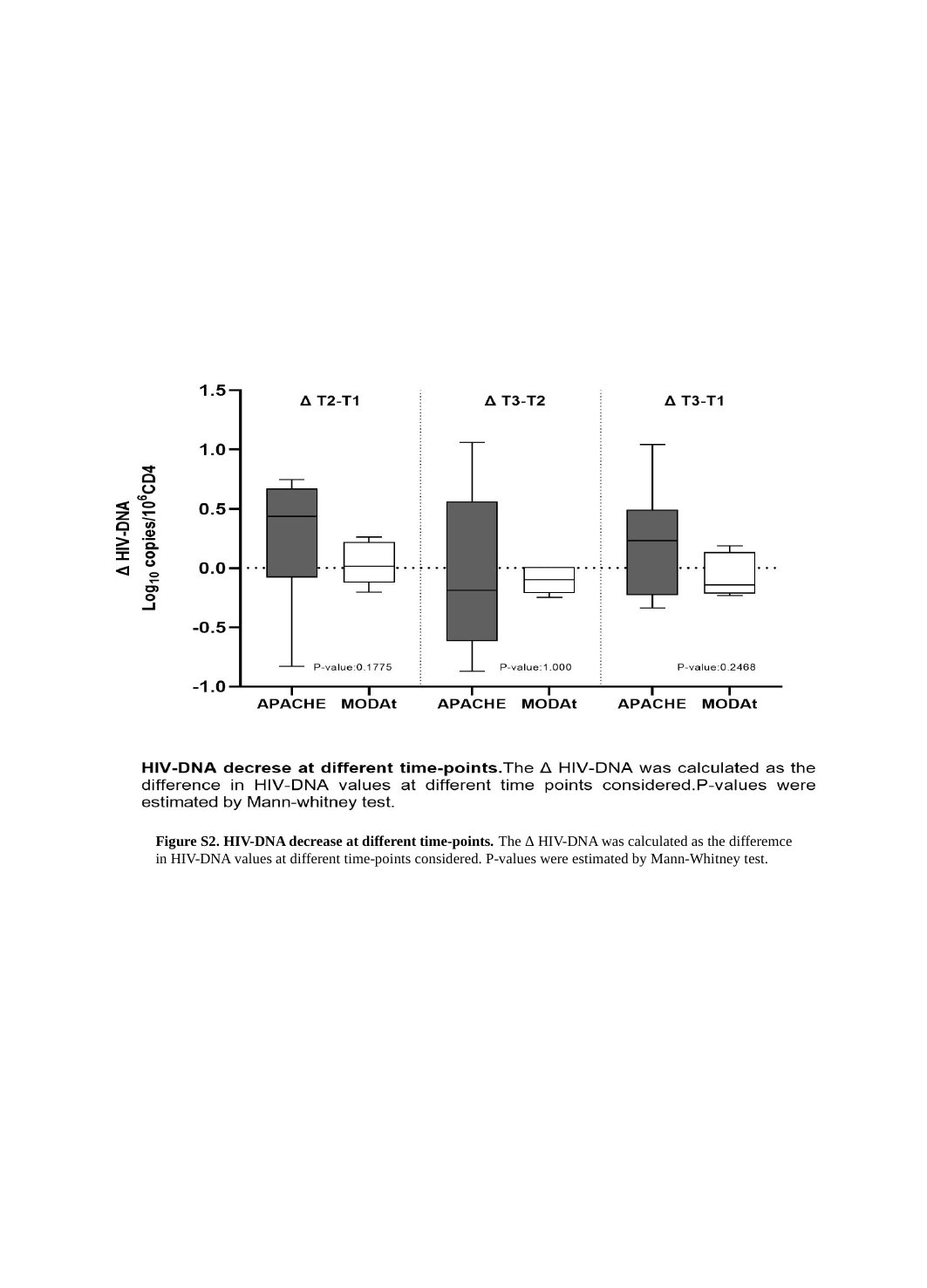

Figure S2. HIV-DNA decrease at different time-points. The Δ HIV-DNA was calculated as the differemce in HIV-DNA values at different time-points considered. P-values were estimated by Mann-Whitney test.
