# Supplementary material for: Impact of Analytical Treatment Interruption on Burden and Diversification of HIV Peripheral Reservoir: A Pilot Study
Source: Viruses. 2021 Jul 19;13(7):1403. doi: 10.3390/v13071403 (PMC8310290; doi:10.3390/v13071403)
Supplement: Supplementary file 1 [file viruses-13-01403-s001.zip › SupplTableS1.pdf]

Table S1. NGS primers used to sequencing HIV-1

Amplicon	Primers	Primer Sequences	HXB2 Nucleotide Positions
**POL Region**			
1	Forward- 1F	5′-AGACAGGCTAATTTTTTAGGGA-3′	2070-2095
	Reverse- 1R	5′-CCAATTATGTTGACAGGTGTAGG-3′	2509-2487
2	Forward -2F	5′CAGGAGCAGATGATACAGTATTAGA-3′	2329-2353
	Reverse-2R	5′-ATGGATTTTCAGGCCCAATTTT-3′	2715-2694
3	Forward-3F	5′-TTAAAGCCAGGAATGGATGG-3′	2583-2602
	Reverse-3R	5′-GGCTCTAAGATTTTTGTCATGC-3'	3058-3057
4	Forward-4F	5′-TGGGAAGTTCAATTAGGAAT-3′	2811-2830
	Reverse-4R	5′-AGGCTGTACTGTCCATT-3′	3278-3262
5	Forward-5F	5′-GCATGACAAAAATCTTAGAGC-3′	2037-3057
	Reverse-5R	5′-TAAGTCTTTTGATGGGTCA-3′	3524-3506
**C2-V3-C3 region**			
1	Forward-S	5′-GTTAAATGGCAGTCTAGCAG-3′	7004-7023
	Reverse-AS1	5′-GAAAAATTCCCCTCCACAATT-3′	7353-7373
